# An Online Acceptance and Mindfulness Intervention for Chronic Pain in Veterans: Development and Protocol for a Pilot Feasibility Randomized Controlled Trial

**DOI:** 10.2196/45887

**Published:** 2023-03-07

**Authors:** Erin D Reilly, Ummul-Kiram Kathawalla, Hannah E Robins, Alicia A Heapy, Timothy P Hogan, Molly E Waring, Karen S Quigley, Charles E Drebing, Timothy Bickmore, Matias Volonte, Megan M Kelly

**Affiliations:** 1 Mental Illness Research, Education, and Clinical Center Veteran Affairs Bedford Healthcare System Department of Veteran Affairs Bedford, MA United States; 2 Department of Psychiatry University of Massachusetts Chan Medical School Worcester, MA United States; 3 Wheelock College of Education & Human Development Boston University Boston, MA United States; 4 Suffolk University Boston, MA United States; 5 Pain Research, Informatics, Multi-morbidities, and Education Center Veterans Affairs Connecticut Healthcare System Department of Veterans Affairs West Haven, CT United States; 6 Yale School of Medicine New Haven, CT United States; 7 Center for Healthcare Organization and Implementation Research Veterans Affairs Bedford Healthcare System Department of Veterans Affairs Bedford, MA United States; 8 Peter O'Donnell Jr School of Public Health University of Texas Southwestern Medical Center Dallas, TX United States; 9 Department of Allied Health Sciences University of Connecticut Storrs, CT United States; 10 Khoury College of Computer Sciences Northeastern University Boston, MA United States; 11 Cheyenne Veterans Affairs Medical Center Department of Veterans Affairs Cheyenne, WY United States

**Keywords:** chronic pain, randomized controlled trial, usability, acceptance and commitment therapy, embodied conversational agent

## Abstract

**Background:**

In the veteran community, chronic pain is particularly prevalent and often debilitating. Until recently, veterans with chronic pain were offered primarily pharmacological intervention options, which rarely suffice and can also have negative health consequences. To better address chronic pain in veterans, the Veterans Health Administration has invested in novel, nonpharmacological behavior interventions that target both pain management and chronic pain–related functional issues. One approach, acceptance and commitment therapy (ACT) for chronic pain, is supported by decades of efficacy evidence for improving pain outcomes; however, ACT can be difficult to obtain owing to issues such as a lack of trained therapists or veterans having difficulty committing to the time and resources needed for the full clinician-led ACT protocol. Given the strong ACT evidence base combined with access limitations, we set out to develop and evaluate Veteran ACT for Chronic Pain (VACT-CP), an online program guided by an embodied conversational agent to improve pain management and functioning.

**Objective:**

The aims of this study are to develop, iteratively refine, and then conduct a pilot feasibility randomized controlled trial (RCT) of a VACT-CP group (n=20) versus a waitlist and treatment-as-usual control group (n=20).

**Methods:**

This research project includes 3 phases. In phase 1, our research team consulted with pain and virtual care experts, developed the preliminary VACT-CP online program, and conducted interviews with providers to obtain their feedback on the intervention. In phase 2, we incorporated feedback from phase 1 into the VACT-CP program and completed initial usability testing with veterans with chronic pain. In phase 3, we are conducting a small pilot feasibility RCT, with the primary outcome being assessment of usability of the VACT-CP system.

**Results:**

This study is currently in phase 3; recruitment for the RCT began in April 2022 and is expected to continue through April 2023. Data collection is expected to be completed by October 2023, with full data analysis completed by late 2023.

**Conclusions:**

The findings from this research project will provide information on the usability of the VACT-CP intervention, as well as secondary outcomes related to treatment satisfaction, pain outcomes (pain-related daily functioning and pain severity), ACT processes (pain acceptance, behavioral avoidance, and valued living), and mental and physical functioning.

**Trial Registration:**

ClinicalTrials.gov NCT03655132; https://clinicaltrials.gov/ct2/show/NCT03655132

**International Registered Report Identifier (IRRID):**

DERR1-10.2196/45887

## Introduction

### Background

Among the most complicated medical conditions to manage, chronic pain refers to pain lasting longer than 3 to 6 months that persists beyond the healing of an initial injury or disease; in fact, it can often last for years or decades [1,2]. Chronic pain is a highly prevalent problem in the United States, with 50 million (20.4%) American adults experiencing daily chronic pain [3]. Military veterans aged ≥20 years are at a particularly high risk because they are 11.4% more likely to have chronic pain than nonveterans of the same age [4]. American veterans, with higher rates of chronic pain and more severe pain than nonveterans, are substantially affected in terms of general functioning and well-being [5-7]. In an effort to reduce chronic pain in this population, pharmacological treatments such as long-term opioid therapy were routinely prescribed and considered standard of care for chronic pain [8]. However, although patients with chronic pain report some relief from short-term use of opioids, there are concerns about long-term opiate use [9], which can lead to substance use and abuse, as well as death [10,11].

To adequately address chronic pain, it is imperative to look beyond pharmacological treatments and encourage the use of behavioral approaches that can enhance self-management of chronic pain. Over the past decade, the Veterans Health Administration (VHA) has strongly promoted a holistic approach to chronic pain treatment that promotes behavioral, therapeutic, and holistic interventions for pain management [12]. Research has shown that addressing factors promoting resilience and psychological well-being can be particularly helpful for individuals with chronic pain [13]. One promising therapeutic framework for pain that emphasizes behavior change and promotes resilience is acceptance and commitment therapy (ACT) for chronic pain, which focuses on mindfulness, value-based goal setting, and psychological flexibility [14]. More than 15 randomized controlled trials (RCTs) to date have shown greater efficacy of ACT for chronic pain than cognitive behavioral therapy, educational and support control groups, medical treatment as usual, or a waitlist control [15,16]. For veterans specifically, ACT for chronic pain has shown effectiveness in improving functioning [17], pain interference, pain-related anxiety, and depression [18].

ACT, similar to other behavioral approaches, requires considerable support from both the health care system (in terms of clinician training and appointment availability) and the patient (in terms of time and effort needed to complete the full treatment protocol). Even when veterans are interested in behavioral interventions for their chronic pain, they may not find it easily or locally accessible. Behavioral treatment for chronic pain requires considerable clinician time and training and, as a result, is less accessible to veterans who are not close to a large Veterans Affairs (VA) medical center. In fact, fewer than half of the veterans diagnosed with chronic pain receive mental health treatment [19], and half of VA facilities do not have any pain-focused behavioral therapy services [20].

To bridge this access gap, technology-assisted pain management interventions are increasingly appealing because remote treatment options can improve the VHA’s ability to reach patients, are cost-effective, and can increase overall clinical impact [21,22]. Web-based behavioral interventions are relatively easy to implement across clinical settings after initial development, compared with in-person interventions, because they do not require training new practitioners to implement a treatment or monitor treatment fidelity [23,24]. In addition, online interventions have the potential to reach many veterans with chronic pain in their homes because 85% of veterans report having access to the internet in their homes [25].

Recent systematic reviews have concluded that technology-delivered ACT is both feasible and effective in managing chronic pain [26]. However, although technology-supported health care interventions are increasing in popularity and are often noninferior to in-person treatment, sustained engagement in these treatments continues to lag [21]. Research has suggested that existing online interventions can increase engagement by adding in more interactive, personalized, and user-tailored feedback [27]. For years, developers of online pain self-management systems have also reported that users become most engaged when the systems provide intelligent responses to the user’s input information related to their pain experiences, concerns, goals, and target behaviors [28]. It should be noted that very few online interventions are able to provide this level of interaction between the user and the system, and even fewer evaluate the impact of personalized features on health outcomes [29] or sustained engagement [23,30]. Consequently, there is still room to improve online pain treatment by developing and testing a more user-centered, interactive, and engaging online pain management experience.

One way to increase engagement with online behavioral interventions is by using embodied conversational agents (ECAs), which are computer-animated characters that simulate interactive dialogue with patients [31]. An ECA can act as a responsive, humanistic, and nonjudgmental coach to guide patients through treatments such as health behavior change interventions, social skills training, and mindfulness-based stress reduction [32-35]. Interacting with ECAs that guide behavior interventions has been associated with positive outcomes such as higher use of stress management skills [33] and healthier eating behaviors in older adults [35]. Clinical trial participants—in particular, individuals with lower levels of health literacy [31]—have also expressed greater satisfaction with interventions that use ECAs than with educational content delivered by other means [33]. By using an ECA as a digital coach for delivering module-based ACT for chronic pain, we aim to provide a fully remote, accessible, and engaging option for at-home pain treatment for veterans with chronic pain.

### Objectives

We are conducting a 3-phase project that will culminate in a pilot feasibility RCT of ECA-delivered ACT for chronic pain. The online program, Veteran ACT for Chronic Pain (VACT-CP), will use a weekly 7-module protocol to guide veterans with chronic pain to decrease behavioral avoidance and increase pain acceptance and valued living to improve their functioning. Using the principles of user-centered design, the aims of this research project are to (1) develop the VACT-CP system with feedback from mental health and other clinical professionals who treat chronic pain; (2) assess the usability of the VACT-CP system via iterative usability testing and development from veteran feedback; and (3) assess the usability of the complete VACT-CP website, as well as describe differences between the VACT-CP group and a waitlist plus treatment-as-usual (WL+TAU) control group on secondary outcomes related to treatment satisfaction, pain outcomes (pain-related daily functioning and pain severity), ACT processes (pain acceptance, behavioral avoidance, and valued living), and mental and physical functioning.

## Methods

### Phase 1: Consultation, Development, and Gathering Provider Feedback

#### Objective

The aim in phase 1 was to gather qualitative feedback on the VACT-CP system from 10 to 12 chronic pain clinical care providers, as well as identify barriers and facilitators for VACT-CP referrals and implementation in the VA Bedford Healthcare System.

#### Participant Recruitment

In phase 1, we conducted semistructured interviews with clinical care providers (n=10) at the VA Bedford Healthcare System, whose work is at least partially related to issues of chronic pain. Participants were recruited using flyers posted around the facility in areas that serve veterans with chronic pain as well as snowball recruitment methods. To ensure a representative mix of clinical care pain providers, we aimed to recruit 2 to 3 psychologists, 2 to 3 nurses, 2 to 3 physicians, and 2 to 3 psychiatrists. The inclusion criteria were as follows: (1) currently working at the VA Bedford Healthcare System or one of its affiliated community-based outpatient clinics in a clinical capacity and (2) currently seeing veterans to assist in their management of chronic pain. The exclusion criterion was any cognitive or physical impairment (eg, auditory or sight issues) that would interfere with aspects of study participation that require using a computer or giving verbal feedback.

#### Provider Feedback Session Procedure

Interested individuals were briefly screened by telephone for eligibility and scheduled for their hour-long session. After providing informed consent, eligible participants completed a quantitative survey and a qualitative interview. The quantitative survey included a brief (5-minute) questionnaire related to their work background and individual demographics. The qualitative interviews were audio recorded for formative assessment of the potential benefits, concerns, and institutional VHA dissemination issues for the VACT-CP intervention using a semistructured interview guide and a *think-aloud* strategy for intervention review.

#### Measures

The demographics questionnaire included basic, nonidentifying information, including participant age, ethnicity, race, and education. The work history survey included questions created specifically for this study related to working with patients who have chronic pain, including the provider’s current position, past experiences as a health or mental health provider specific to working with patients with chronic pain, length of time in their current position, past specialized training on treating chronic pain, and information related to making any prior referrals to online or technology-mediated health interventions or resources. The qualitative interview included open-ended questions related to palatability of the intervention; feasibility of engagement with veterans with chronic pain; interest in, or potential concerns regarding, referring veterans to such a program; and reactions to a short walk-through of the intervention. Questions about palatability were based on the “organizational perspective” components of the Practical, Robust Implementation and Sustainability Model [36] (Multimedia Appendix 1).

#### Data Analysis Plan

After data collection, we used provider feedback to (1) understand the acceptability and potential usability of the platform in other settings (eg, at the veteran’s home), (2) examine provider attitudes toward the VACT-CP system, (3) address any technological or system-specific concerns, and (4) modify potential plans for the future VACT-CP phase 2 usability assessment. Modifications to the interface and protocol were based on (1) acceptability and feasibility of the VACT-CP platform and potential treatment components and (2) qualitative feedback obtained from providers. Individual qualitative interviews will be analyzed at the end of the full study via the consensual qualitative research (CQR) approach. The initial codebook will be used by 2 to 3 coders throughout the data analysis process to foster multiple perspectives until consensus is reached among the coders about the meaning of the data. In addition, at least 1 auditor will check the work of the primary team of coders and serve to minimize potential bias.

We will report the frequency of emerging domains and themes. Themes will then be categorized according to the following CQR groupings: general (include all or all but one of the cases), typical (include more than half of the cases up to the cutoff for general), and variant (at least 2 cases up to the cutoff for typical). We will use these data to discuss broader scientific and implementation-related barriers and facilitators for interventions for patients with chronic pain within the VA system. Full data for this phase will be analyzed at the end of the completed study.

### Phase 2: Veteran Usability Assessment

#### Objective

The objective in phase 2 was to conduct an iterative usability assessment by pilot-testing VACT-CP components, including the format, digital ECA guide (*Coach Anne*), and system via field-based iterative usability testing (3 waves; n=4-5 per testing wave, total goal: N=12-15).

#### Participant Recruitment

Veteran participants for phase 2 were recruited through a variety of methods, including through providers who shared information about the study with potential participants, posting flyers throughout the hospital, and sending mailers to veterans who received treatment for chronic pain within the past 6 months as identified via a medical chart review. The inclusion criteria were as follows: (1) veteran aged ≥18 years; (2) current diagnosis of noncancer chronic pain; (3) competent to provide written informed consent; and (4) has a working, high-speed wireless internet connection at home or is willing to access the website at the VA Bedford Healthcare System using a provided laptop computer in a secure space. The exclusion criterion was any cognitive or physical impairment (eg, auditory or sight issues) that would interfere with aspects of study participation that require using a computer or giving verbal feedback.

#### Usability Testing Procedure

We aimed to have 3 different usability testing waves, with at least 1 week between each wave to allow enough time to iteratively address any functionality issues or concerns with the user interface. The choice of 4 to 5 participants per wave was based on the finding that approximately 80% of usability problems can be detected using a sample of 4 to 5 participants [37]. Usability sessions were conducted remotely using a secure videoconferencing platform or in person at the Bedford VA medical center, if preferred by the participant. The goals of phase 2 were to (1) obtain further feedback on the VACT-CP treatment by gathering information from veterans via usability testing designed to assess interest in, satisfaction regarding, and usability of the online VACT-CP treatment; and (2) iteratively address any technological concerns. We first completed a telephone screen to assess whether veterans were eligible according to our inclusion and exclusion criteria. After the intake assessment, eligible participants were scheduled for a single 2-hour usability testing session. After providing verbal informed consent, participants completed a pre–usability testing survey battery. Subsequently, they were provided with a hyperlink and their precreated account information and asked to complete the first and second modules (combined) of the VACT-CP system, guided by Coach Anne. Participants were instructed to *think aloud* as they completed the module and provide their first reactions to the program. VACT-CP usability was evaluated along four dimensions: (1) usefulness: whether users can successfully complete designated tasks on the website, (2) effectiveness: whether users can accomplish tasks quickly and easily, (3) learnability: whether users meet predetermined site navigation goals within a specified period of time, and (4) satisfaction: how users feel about the website. Objective measures included usability scales related to content, perceived usefulness, visual appeal, and overall usability of the system. A qualitative interview was conducted after the think-aloud session to gather additional feedback, identify which aspects of the program were the most or least helpful, and review any suggested changes. Participants received a US $40 gift card for their 2-hour digital research session.

#### Measures

##### Demographics, Functional Health, and Pain Measures

Demographic measures included questions on pain type and duration, age, gender identity, race, ethnicity, and education level. Additional measures will include the Patient Health Questionnaire-9 [38] and Posttraumatic Stress Disorder Checklist [39], as well as pain interference (Brief Pain Inventory) [40] and the Veterans RAND 36-Item Health Survey (VR-36) [41]. These measures will be used to describe the demographic and health-related characteristics of the sample.

##### Media and Technology Use and Attitudes Scale

The Media and Technology Use and Attitudes Scale [42] is a 16-item measure of attitudes toward technology with a 10-point frequency scale, from 0=never to 10=all the time, that assesses comfort level, attitudes toward new technology, and use patterns for mobile technology. This scale is completed before the VACT-CP interaction session and will be used to describe the technology attitudes and perceptions of the sample.

##### System Usability Scale

The System Usability Scale (SUS) [43] is a 10-item measure that uses a 5-point Likert scale from 1=strongly disagree to 5=strongly agree to assess the usability of a technology. The SUS, a globally used scale, generates a subjective evaluation score to determine whether the technology system in its current form is sufficiently usable. An SUS score of >68 is regarded as above average usability, and an SUS score of >80 is regarded as high; when people rate a technology product with an SUS score of >80, they are likely to recommend it to friends [43].

##### Intrinsic Motivation, Usability, and Perceived Usefulness Scales

The usability survey items are adapted from an existing set of usability questions to make the questions specific to the VACT-CP treatment and are informed by the Unified Theory of Acceptance of Technology [44,45] and the Wilson and Lankton [46] model, which combines the Technology Acceptance Model [47] and motivational model of technology acceptance [48]. The survey items assess the following factors: intrinsic motivation to use the VACT-CP system, perceived ease of use, perceived usefulness, and behavioral intention to use the system. Each item is assessed using a 5-point Likert scale, and participants are asked to indicate their level of agreement with each item on a scale from 1=strongly disagree to 5=strongly agree. Scale scores (agreement ratings averaged across items for each construct) are then calculated for each of the following constructs: intrinsic motivation, ease of use, perceived usefulness, and behavioral intention to use the VACT-CP system.

##### Qualitative Combined Usability and Contextual Interview

Participants were observed while working with the VACT-CP system, including being video recorded and asked questions by an interviewer. The interview guide (Multimedia Appendix 2) includes specific questions related to the different parts of the system (eg, the digital coach design, structure of the modules, and feedback system). In addition, interviewers query participants for feedback or information when participants seem to be confused or report difficulties with the system (eg, What are you trying to get to from this page? and What could be better about how this is set up?). All questions are open-ended and ask about multiple areas of usability and content, such as problems accessing and using the VACT-CP treatment, the perceived usefulness or importance of VACT-CP areas of treatment, and whether the participant would recommend this treatment to a fellow veteran.

##### Intervention Revision

Between each wave of testing, information specific to usability issues was gathered and analyzed to address any technological issues and revise content. Modifications to the website, module structure, and overall VACT-CP website framework were made between each wave based upon acceptability and feasibility of the online treatment and qualitative feedback obtained from patients.

#### Data Analysis Plan

Similar to phase 1, the video recordings and notes made during the qualitative portion of user testing will be transcribed and analyzed using Excel (Microsoft Corp). The quantitative data from the SUS and usability survey will be used to obtain scale scores that describe each veteran’s general perceptions and opinions of the usability and content of the VACT-CP system. This feedback will then be reported in conjunction with qualitative usability and acceptability data, which will be analyzed using the modified CQR approach also planned for in phase 1. In phase 2, the themes generated by these qualitative data will be used to identify user system issues, general trends in use of the website, content issues, how well users can complete an assigned task on the VACT-CP system, and where they are encountering problems. Using this method, we will classify all general comments regarding usability as necessary to include in iterative development and site changes, whereas variant and typical usability concerns will be discussed within the research group to decide upon potential ways to incorporate this feedback into future intervention refinement. Full analysis of these data is ongoing, although initial general usability issues have been resolved, and suggestions for website refinement have already been integrated into the VACT-CP system.

### Phase 3: Small Pilot Feasibility RCT

#### Objective

We will conduct a stage IB pilot feasibility RCT to assess the usability, feasibility, and acceptability of the VACT-CP system compared with a WL+TAU control group. This will include (1) evaluating the usability of the VACT-CP system; (2) assessing the relative feasibility and acceptability of the VACT-CP intervention procedure and WL+TAU control, including ease of recruitment, retention in each condition, treatment receptivity, attrition and retention in each condition, sustained VACT-CP website use, and the assessment process; and (3) describing changes in pain acceptance, valued living, mental and physical functioning, pain-related interference in daily functioning, and behavioral avoidance for each group. This trial has been registered on ClinicalTrials.gov (NCT03655132).

#### Participant Recruitment

We aim to recruit and complete baseline assessments for up to 60 participants over 15 months to allow for the required 40 participants to be screened as eligible and successfully randomized. Participants will be screened based on the inclusion and exclusion criteria. The inclusion criteria will include (1) veteran aged ≥18 years; (2) current diagnosis of noncancer chronic pain (as noted in the phase 2 inclusion criteria); (3) has a working, high-speed wireless internet connection at home or is willing to access the website over the 7-week intervention at the VA Bedford Healthcare System using a provided laptop computer in a secure space; and (4) competent to provide written informed consent. The exclusion criteria will include (1) any current or lifetime Diagnostic and Statistical Manual of Mental Disorders, Fifth Edition (DSM-5), psychotic disorder; (2) current or recent (within 1 month of study entry) DSM-5 alcohol or drug use disorder; (3) current use of any other chronic pain–related behavioral or psychological treatment; (4) any cognitive impairment that would interfere with study participation; (5) clinically significant suicidality within the past year; (6) presence of any clinical features requiring a higher level of care (inpatient or partial hospital treatment); and (7) any cognitive or physical impairment that would interfere with aspects of study participation that require using a computer and providing feedback. Participants with >1 pain diagnosis or comorbid mental health diagnoses other than those already listed will not be excluded.

#### RCT Procedures

Potential participants will be prescreened by telephone to determine whether study eligibility criteria are met. Individuals who remain eligible after the telephone prescreen will be scheduled for a baseline session during which staff will obtain informed consent and confirm clinical eligibility through the Structured Clinical Interview for DSM-5 to assess for potential psychosis, substance use disorders (SUDs), or clinically significant suicidality. Eligible participants will complete self-report measures via an online or mailed paper survey, and after completion of the survey, they will be randomized in a 1:1 ratio into 4 randomly permuted blocks (10 per block) to either the VACT-CP condition or the WL+TAU control condition. Veterans randomized to the VACT-CP condition will receive 7 online weekly modules to be accessed using a desktop or laptop computer within a secure space at the VA Bedford Healthcare System. Veterans in the WL+TAU control group will be provided with a list of common pain resources and encouraged to connect to their local VA pain clinic if interested in pain management care. All participants will complete the 4 surveys. At the conclusion of the study, participants in the WL+TAU condition will be provided with the VACT-CP website as an optional pain management resource, but they will not be asked to use it and will not be followed as research participants. Participants will be compensated US $60 for the baseline assessment (approximately 1 hour), US $40 at the midpoint (week 3; approximately 45 minutes), US $60 at the end of treatment (week 7; approximately 1 hour), and US $40 for the week 11 (1-month follow-up; approximately 40 minutes) for a total possible compensation of US $200.

#### VACT-CP Treatment Content and Structure

Table 1 shows the treatment components that will be emphasized in the VACT-CP program based on previous manualized ACT for chronic pain treatments and workbooks [15,49-52].

**Table 1:** Veteran Acceptance and Commitment Therapy for Chronic Pain (VACT-CP) timeline and intervention description.

Timeline	Module focus	Intervention components
Week 1	Introduction to the VACT-CP program	The introductory module assesses the nature of the veteran’s pain, past treatments, and experience with acceptance and mindfulness. Coach Anne provides information on ACT^a^ and assesses the veteran’s values and goals to help them set a weekly goal.
Week 2	Behavioral change and triggers	This module focuses more broadly on pain psychoeducation and pain management. Veterans complete exercises on dealing with cognitive barriers with the goal of changing their own self-identified obstructions to living a meaningful life.
Week 3	Acceptance and mindfulness	This module uses metaphors and a veteran narrative story to explain and explore concepts of acceptance and mindfulness, and the veteran completes an acceptance exercise linked to previously identified values.
Week 4	Cognitive defusion	This module focuses on the ACT concept of cognitive defusion using psychoeducation about the nature of language and cognitive barriers to valued living. Metaphors, mindfulness, and ACT exercises are included.
Week 5	Willingness	This module explains the ACT concept of willingness and discusses willingness to experience discomfort as a means to pursuing important valued living goals. The veteran creates and applies a willingness hierarchy to identify a goal for the week that embodies values-driven willingness.
Week 6	Committed action	This module focuses on the ACT concept of committed action. Coach Anne helps the user to identify personal barriers to committed action and strategies for dealing with these perceived barriers.
Week 7	Valued living wrap-up	The final session provides an overview of ACT processes, a review of goals achieved, and reinforces progress made during the program. This session ends with planning for the “lifelong assignment” of fully engaging in one’s life.
Additional modules	Mindfulness exercises module	This module introduces the topic of mindfulness and provides a series of questions to route users to use exercises that align with their goal for the mindfulness experience. This module is accessible any time after completing the first module.
Additional modules	Additional resources	This module provides additional information, links, and resources for care options within the VHA^b^ for pain, psychiatric conditions, and physical health management.

^a^ACT: acceptance and commitment therapy.

^b^VHA: Veterans Health Administration.

**Figure 1:**
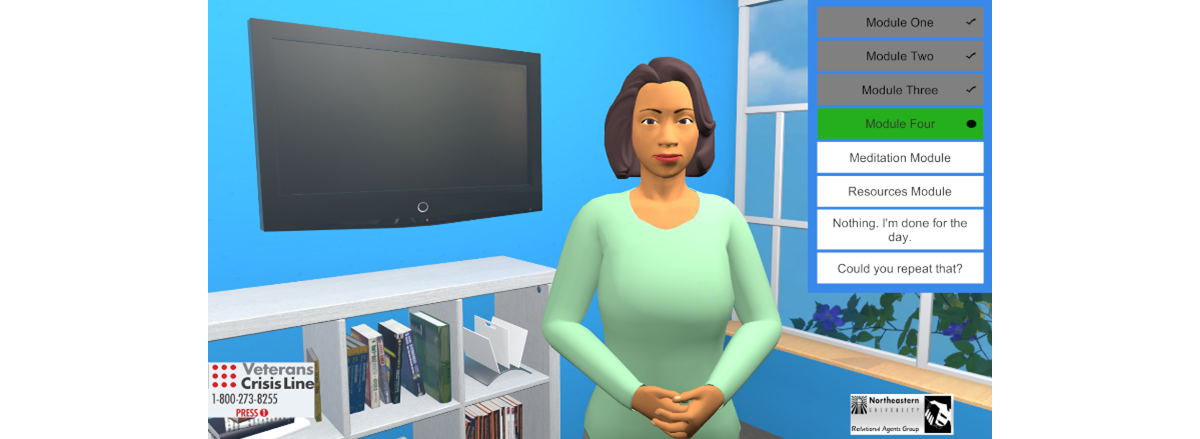
Screenshot of the Veteran Acceptance and Commitment Therapy for Chronic Pain website program.

The 7 online modules that participants will receive as weekly sessions feature Coach Anne as a digital treatment guide (Figure 1). The website is currently only accessible on desktop and laptop computers; it is not available as a mobile-enabled website for mobile phones. Seven modules were chosen, consistent with reviews of module-based online ACT that suggested that 7 to 8 modules are sufficient to allow users to experience all aspects of ACT [26]. All content is presented interactively through text-based 2-way conversations with Coach Anne. Veterans hear what Coach Anne *says* and can respond to her queries using forced-choice text options that will trigger different responses from Coach Anne as the conversation progresses, allowing the system to responsively interact in a personalized manner with the veteran (for an example of our video introduction, refer to Multimedia Appendix 3). The modules also use videos (eg, veteran narratives and metaphors), in-module assessments (eg, values assessments), and interactive exercises for goal setting. Each week, the user is reminded via an encrypted email to complete their next online module.

Once a module has been completed, the next week’s module opens 5 days later to allow for more flexible completion of the 7 sessions. Thus, the shortest duration over which a user can complete the VACT-CP protocol is 35 days. The system prevents the user from completing the next week’s module immediately because they must wait 5 days for the next module to open. This was incorporated to provide the user with several days to meet self-created goals, practice skills, and use other resources provided through the website as desired. Participants will be contacted via telephone at week 3 and week 5 to check in on any usability issues and potential concerns, as well as to assess for any possible risk related to mood, physical health, or suicidal ideation. Users can access the website for their entire participation time (11 weeks).

#### Measures

##### Demographics, Functional Health, and Pain Measures

We will use many of the same surveys from phase 2 in our phase 3 data collection, including the Patient Health Questionnaire-9, Posttraumatic Stress Disorder Checklist, Brief Pain Inventory, VR-36, Intrinsic Motivation and Perceived Usefulness Scales [53], and SUS [54]. In addition, we will collect the information encapsulated in the following sections.

##### Feasibility and Acceptability Measures

Feasibility outcomes will include information on recruitment rates, retention rates within each condition, completion rates, how often each veteran accesses the website, and any reported problems with the website. This information will be collected through open-ended survey items, website data, and through use of a semistructured post–VACT-CP qualitative interview (Multimedia Appendix 4).

##### Pain-Related Daily Functioning

The Pain Outcomes Questionnaire-Short Form [55] is a 19-item inventory that assesses a patient’s ability to engage in functional activities related to daily living that may be affected by pain interference. Each item is rated on an 11-point (0-10) Likert-type scale and yields an overall average score of all 20 items, resulting in a total score between 0 and 10. In addition, the scale contains a pain numeric rating scale item and 5 subscales measuring (1) activities of daily living, (2) negative affect, (3) mobility, (4) vitality, and (5) fear.

##### Pain Acceptance

The Chronic Pain Acceptance Questionnaire-Revised [56] is 20-item survey measuring recognition that pain does not prevent one from living a valued life. The questionnaire consists of two subscales: (1) activity engagement (11 items), or the degree to which one engages in life activities regardless of the pain; and (2) pain willingness (9 items), or an individual’s willingness to experience pain. In addition, a total score is created by summing all items [57]. The scales have shown adequate reliability and validity and are substantially related to the other measures of patient functioning [58].

##### Valued Living

The Chronic Pain Values Inventory [59] is a 12-item self-report measure of the extent to which a patient is living in accordance with their values in areas such as work, health, and family, and which is related to lower perceived disability and pain-related anxiety, as well as greater reported patient functioning, even in the context of high levels of pain.

##### Behavioral Avoidance

The Multidimensional Experiential Avoidance Questionnaire (MEAQ) [60] is a 62-item self-report measure of experiential avoidance, including subscales on behavioral avoidance, distress aversion, procrastination, distraction and suppression, repression and denial, and distress endurance. The MEAQ has shown good internal consistency across all subscales, with Cronbach *α* averaging .83 across multiple samples [60,61].

##### Patient Satisfaction With Treatment

The Client Satisfaction Questionnaire-8 [62] is an 8-item scale that measures global treatment satisfaction based on perceived treatment quality and effectiveness of the intervention. This scale has been used at mental health and other health centers and has acceptable internal consistency (Cronbach *α*=.83-.93).

#### Planned Analyses

##### Sample Size Considerations

Consistent with the recommended stage model for development of behavioral therapies, the aim of this stage IB pilot feasibility RCT is to inform future treatment development and evaluation. Given that prior investigations of ACT have reported effect sizes in the large-to-medium range, we expect that the VACT-CP intervention will also produce at least a medium treatment effect. To have a power of 0.80 to detect differences at a 2-tailed *α* level of .05, a medium effect would require 125 participants. This was beyond the objective and scope of this preliminary stage IB pilot feasibility RCT. A total sample size of 40 (n=20, 50%, per group) is consistent with the recommendation by Rounsaville et al [63] of 15 to 30 participants per condition for stage IB behavioral treatment development. For phase 3, the total goal for enrollment and randomization is 40 participants (n=20, 50%, per group) for the 7-week VACT-CP intervention and WL+TAU control groups. Assuming an attrition rate of 30% after randomization based on past attrition rates for online behavior interventions [30], we plan to enroll up to 60 participants to meet our goal of 40 successfully randomized participants.

##### Primary Analyses

Usability of the VACT-CP system as assessed through the SUS is the primary outcome for the RCT. Scores for those in the VACT-CP group will be aggregated and described according to these metrics, with an SUS score of >68 interpreted as above average and a score of >80 as high and reflective of a system that participants are likely to recommend to others [64]. We will also describe changes on the Usability Survey Scales from before to after the intervention. Additional feasibility and usability data will be provided by the semistructured qualitative interviews and tracking of any telephone calls or emails to research staff with concerns. Similar to phases 1 and 2, phase 3 analysis of postintervention interview transcripts will use CQR techniques (eg, coding for and extracting major themes) to assess both barriers and facilitators for use of the VACT-CP system, as well as individual demographic user factors that affect use and perceived usefulness. Finally, we will assess intervention feasibility by measuring the proportion of individuals who successfully complete at least 5 (71%) VACT-CP modules of the total 7, which would be slightly above the mean percentage of completed sessions observed in previous online behavior interventions [30].

##### Secondary Analyses

Secondary exploratory analyses will describe satisfaction with the VACT-CP system and the potential impact of the intervention on treatment outcomes related to pain-related interference in daily functioning and severity (Pain Outcomes Questionnaire-Short Form), pain acceptance (Chronic Pain Acceptance Questionnaire-Revised), behavioral avoidance (MEAQ), valued living (Chronic Pain Values Inventory), and mental and physical functioning (VR-36 Mental Component Score and VR-36 Physical Component Score). On the basis of the intent-to-treat principle, all participants enrolled and successfully randomized will be included in these analyses, and the principal investigator will conduct preliminarily analyses and descriptively report on the pilot RCT outcomes of the VACT-CP intervention to inform further refinements to it. Descriptive measures (means and SDs) will be reported for all outcomes of interest. We anticipate that, for VACT-CP participants, we will observe increases in the mean scores for pain acceptance, valued living, and mental and physical functioning, as well as a decrease in pain-related interference in daily functioning and behavioral avoidance. In addition, we predict that there will be small-to-no changes in mean pain severity levels, given research suggesting that behavior interventions do not change pain levels for participants with chronic pain. Given imprecise estimations owing to the pilot’s small sample size and a lack of power, we will not report estimates of effect sizes for these specific predictions. However, we will report descriptive outcomes (means and SDs) between before and after the intervention as well as between the groups and use nonparametric testing to report changes in secondary outcomes at the individual and group levels (pain acceptance, valued living, and mental and physical functioning, as well as pain-related interference in daily functioning and behavioral avoidance).

### Ethics Approval

All 3 phases of this study were approved by the institutional review board of the VA Bedford Healthcare System Research & Development Committee in Bedford, Massachusetts, United States (1598754), with initial approval granted in September 2018.

## Results

Participant recruitment for phase 3 began in April 2022 and is currently ongoing. We expect that recruitment will be complete by April 2023, data collection completed by October 2023, and primary data analyses completed by late 2023.

## Discussion

### Potential Applications

Online programs and at-home treatments for chronic pain are increasingly popular, but there is room to improve the breadth and efficacy of remote treatment options and to create a more engaging user experience. The intended outcome product, online VACT-CP, requires this multiphase project to successfully apply user-centered design best practices in developing our proposed intervention. Good usability and acceptability research should highlight the biggest needs for the proposed end product, but we cannot know all requirements before beginning the design processes. Consequently, by having multiple stages of feedback across multiple stakeholder groups, we will be able to develop and continually improve on the initial proposed VACT-CP system. Given the existing research base for our preliminary system, we believe that this online, ECA-delivered intervention may be a promising treatment option for chronic pain for the veteran population.

### Limitations and Future Directions

This study includes limitations specifically related to its small sample size and, therefore, generalizability. However, our proposed sample size aligns with prior research on the number of participants needed to assess usability and feasibility. In addition, the format of the website requires a person to have access to a laptop or desktop computer because the current website is not mobile-device enabled. However, as a proof-of-concept design, successful findings from this study could be leveraged to further revise the site to allow for mobile options. In addition, excluding participants with an active SUD is a limitation. This comorbidity is common among veterans with chronic pain, and this online intervention could potentially assist in both their pain management and substance use owing to the transdiagnostic nature of ACT interventions. However, for this preliminary study, such an exclusion is warranted to ensure that participants are medically stable and not reporting clinical symptoms that would require a higher level of care. In the future, it would be useful to allow participants with an active SUD to participate if interested and as part of their SUD treatment. Finally, this study will include a majority of veterans living in 1 region of the country. Further testing could include multiple sites, including other VA medical centers that serve more rural populations that would potentially benefit from efficacious remotely delivered treatments for chronic pain.

### Conclusions

This research project has the potential to create an easily disseminated and highly accessible pain treatment option, VACT-CP. In addition, the online intervention is potentially more engaging, accessible, and cost-effective than other existing versions of ACT for chronic pain. An interdisciplinary research team, comprising experts in ACT interventions, chronic pain, and technology-assisted health care assessment and delivery, will create and refine the VACT-CP system. Multiple stages of testing with veterans will further refine the intervention as well. Overall, it is expected that this 3-phase research project will lead to the development of a veteran-centered pain management website that helps users to better manage pain-related symptoms and focus on valued living domains. This study will provide preliminary feasibility data on whether the VACT-CP system is usable and acceptable. The final online program would allow all veterans with a home computer and internet access the option of an engaging, user-centered pain intervention. The VACT-CP system may also be particularly well received by, and of interest to, patients with chronic pain, given the lasting impact of COVID-19 on increased acceptance of, and interest in, remotely delivered interventions. After completion of this project, we will evaluate whether a future study is warranted in the form of a larger-scale VACT-CP efficacy trial to investigate whether the intervention improves pain-related functioning and quality of life.

## Appendix

Multimedia Appendix 1 Phase 1: Veteran Acceptance and Commitment Therapy for Chronic Pain provider interview protocol.

Multimedia Appendix 2 Phase 2: Usability and contextual interview protocol.

Multimedia Appendix 3 Introductory tutorial video of the Veteran Acceptance and Commitment Therapy for Chronic Pain online program.

Multimedia Appendix 4 Phase 3: Veteran Acceptance and Commitment Therapy for Chronic Pain postintervention feedback interview.

Multimedia Appendix 5 Summary statement by Career Development Program Panel II - Rehabilitation Research and Development Parent IRG - Office of Research & Development (National Institutes of Health, USA).

## Abbreviations

ACT: acceptance and commitment therapy
CQR: consensual qualitative research
DSM-5: Diagnostic and Statistical Manual of Mental Disorders, Fifth Edition
ECA: embodied conversational agent
MEAQ: Multidimensional Experiential Avoidance Questionnaire
RCT: randomized controlled trial
SUD: substance use disorder
SUS: System Usability Scale
VA: Veterans Affairs
VACT-CP: Veteran Acceptance and Commitment Therapy for Chronic Pain
VHA: Veterans Health Administration
VR-36: Veterans RAND 36-Item Health Survey
WL+TAU: waitlist plus treatment-as-usual

## Data Availability

Owing to privacy and ethical considerations, the final data sets underlying publications resulting from this research will not be publicly shared outside of the Department of Veterans Affairs. On request and with principal investigator (EDR) permission, a deidentified, anonymized data set will be created and shared pursuant to a data use agreement, appropriately limiting the use of the data set and prohibiting the recipient from identifying or reidentifying (or taking steps to identify or reidentify) any individual whose data are included in the data set.
