# Supplementary material for: An Online Acceptance and Mindfulness Intervention for Chronic Pain in Veterans: Development and Protocol for a Pilot Feasibility Randomized Controlled Trial
Source: JMIR Res Protoc. 2023 Mar 7;12:e45887. doi: 10.2196/45887 (PMC10031449; doi:10.2196/45887)
Supplement: Multimedia Appendix 1 [file resprot_v12i1e45887_app1.pdf]

PHASE 1: VACT-CP PROVIDER INTERVIEW PROTOCOL

PROVIDER INTERVIEW GOALS	
Part 1. Descriptive questions: demographics, history of clinical work with veterans, past experience with veteran referrals for chronic pain and inclusion of technology (i.e., mobile apps.) in treatment.	
Part 2. Present the participant with a walk-through of the intervention and user interface options. Using a "think aloud" strategy, ask them to give their immediate thoughts out loud as they interact with the system. Ask clarifying questions as appropriate.	
Part 3. Open-ended questions related to palatability from a current provider or as a potential referral source perspective will be asked.	
**PROVIDER ELEMENT**	**OPEN-ENDED QUESTIONS FOR PROVIDERS**
**General**	What do you think about online programming for veteran support for chronic pain in general? What are your overall thoughts on the purpose of the VACT-CP intervention?
**Patient centeredness**	How do you think Veterans could benefit from the program online?
**Provides patient choices**	For what reasons might you refer veterans to this program as an option for treatment? Are there any veterans that you would not refer to this type of program because you don't think they would engage or benefit?
**Addresses patient barriers**	What might help you feel comfortable referring a veteran to this program? What might be some barriers to referring a veteran to this program?
**Additional program elements**	Do you think the provided handout on this intervention would give you enough information to refer veterans to this intervention? What would you change about these materials?
**Burden/Barriers**	What do you see as potential barriers or concerns for Veterans who might sign up for this online treatment?
**Feedback of results**	Would you be interested in hearing about VACT-CP results after the study ends?

General Interview Procedure

- Be sure that that audio recorder and VACT-CP prototype (on a laptop or computer) are already in the room and are working effectively.
- **Remind the participant that the entire session will take about one hour.** Obtain consent, and have the participant fill out the demographic and work-history questions.
- Explain that we will first take some time exploring their past treatment of chronic pain with Veterans, their concerns regarding Veterans managing their chronic pain at home, and their technology use.
- Following that, we will demo some of the intervention components we are designing for VACT-CP to deliver, to get their feedback on the interactions, design, and overall opinions. After showing the website prototype images, we will ask some follow-up questions as well.
- Emphasize that their input is vital in helping to identify and prioritize needs, create solutions, and planning for services and future research pilots.

<u>Interview Questions</u>

First, I have some questions about your past experience in treating chronic pain with Veterans.

- How do you typically help your patients manage their chronic pain?
  - What interventions have you used in the past to help your VA patients manage chronic pain?
  - Have you suggested chronic pain treatments that were non-medical?
  - What are the services you typically refer Veterans to when they have chronic pain issues?
    - i.e. Chiropractors, therapy, self-help groups, etc.
    - Internal to the VA? External resources?

- What have you found to be the most helpful or useful thing that you have learned about treating chronic pain?

- What challenges do you have helping your patients manage their chronic pain?
  - What methods have you found the least helpful in treating chronic pain?

- What do you wish Veterans would do at home to manage their chronic pain?
  - What are their biggest struggles or barriers at home to managing pain?
  - What do Veterans struggle with most at home?
  - What do you wish you or the VA could provide to help Veterans at home?

Clinician Endorsements of Technologies

Now, I have some questions your use of technology, and about the kind of technology you have incorporated into helping Veterans treat their pain, or not.

- How comfortable do you **personally feel** with technology?
  - Have you personally ever used a website or app to assist in managing your health?

- Have you ever referred Veterans to any technology (i.e., mobile apps.) to help them treat their <u>chronic pain</u>?
  - [y] What specifically have you referred Veterans to use for their pain?
  - [n] Why not?

- Do you ever suggest apps to Veterans for other health purposes? (e.g., tracking weight, exercise, diet, medication tracking, or other health behaviors)
    - [yes] Can you tell me about some things you've suggested for health-related apps before?
        - What app (or tech) do you suggest the most for health purposes?
    - [no] Would you consider referring Veterans to mobile apps for health issues? Why or why not?

- Think about the last piece of technology that you referred a Veteran to use. What was it? What was the Veteran's experience?

- What do you think about online programming or apps for Veteran support for chronic pain in general?

We are currently working with a team to create an online therapeutic resource for Veterans with chronic pain. Specifically, we will be creating an online intervention that has 8 sessions to help Veterans manage their chronic pain, and continue to live their lives in ways that are aligned with their values. This would be provided to Veterans for free, and the website would feature a virtual therapist to walk them through each treatment session. We would like your feedback on our ideas for this intervention.

- In what ways might you feel comfortable referring a Veteran to this online program to help them manage their chronic pain?

- What might be some barriers to referring a Veteran to this online program?
    - Are there any Veterans that you would not refer to this type of program because you don't think they would engage or benefit?
    - Would you be concerned about the Veterans safety if they used a website as a supplement to other treatments for chronic pain? Why or why not?
    - What do you see as potential barriers to referral, or for Veterans to sign up for this online treatment? What might concern you about suggesting that a Veteran use VACT-CP?

- What would you need to trust that the site was secure and private?
- Imagine that you referred a Veteran to an online intervention for chronic pain. What, if anything, would you want the program to provide to you as follow-up?
    - An update on if they are using it? A report on their progress or status? Assessment information?

VACT-CP Elements

Now, we're going to show you some of the elements we are designing for our online website, which Veterans would use at home. Feel free to discuss out loud your impressions while I show you the website prototype, including what you like, do not like, or would change. We want to get your opinion on different parts of each, so I will ask you questions after and sometimes during each demo. [go through module sheet, handout of avatars and backgrounds]

- General questions to ask after the slideshow/website walk-through.
  - How do you feel about the room?
  - How did you like the visuals and graphics for the virtual therapy guide?
  - Would you have any concerns about Veterans doing skills/exercises at home with the virtual therapist?

Following VACT-CP Review

Thank you for your feedback so far; the last thing we want to do is get some of your general and specific thoughts on the website as a whole, and its potential for future use. First….

- What are your overall thoughts on VACT-CP?
  - What information would you want from us, perhaps in the form of a handout, that you would want to be able to have the information you need to refer people to this program?
- What (else) would you suggest to make it easier to use (more user-friendly) or more inviting to use?
  - [possible areas: privacy, data storage, general technology use concerns, etc.]
- Do you think the provided handout on this intervention would give you enough information to refer veterans to this intervention? What would you change about these materials?
- Would you be interested in hearing about VACT-CP results after the study ends?
