# Supplementary material for: An Online Acceptance and Mindfulness Intervention for Chronic Pain in Veterans: Development and Protocol for a Pilot Feasibility Randomized Controlled Trial
Source: JMIR Res Protoc. 2023 Mar 7;12:e45887. doi: 10.2196/45887 (PMC10031449; doi:10.2196/45887)
Supplement: Multimedia Appendix 2 [file resprot_v12i1e45887_app2.pdf]

PHASE 2: USABILITY AND CONTEXTUAL INTERVIEW PROTOCOL

GENERAL PROCEDURE

- Be sure that that VACT-CP website and Skype/Cisco website are ready and are working effectively. Make sure the video is set up on the computer.
- Remind the Veteran that the entire session will take about 2 hours. Obtain verbal consent, and have the Veteran fill out the surveys packet #1 online or in-person.
- Explain that we will first take a few minutes identifying their concerns in managing their chronic pain at home and their technology use.
- Following that, we will demo some of the intervention components we are designing for VACT-CP website to deliver, to get their feedback on the interactions, design, and overall opinions. After showing what VACT-CP website can do and giving you the opportunity to go through a Module, we will ask some follow-up questions as well.
- Emphasize that their input is vital in helping to identify and prioritize needs, create solutions, and planning for services and future research pilots.

Pre-Demo Interview Questions

First, I have some questions about your past chronic pain treatments.

- What have you done previously to help manage your chronic pain?

- Have you engaged in past chronic pain treatment that was non-medical?
    - *What have you found to be the most helpful or useful think that you have learned in your chronic pain treatment?*
    - *What did you find least helpful?*

- Do you sometimes have problems when trying to manage your chronic pain at home?
    - *What are you biggest struggles or barriers at home to managing your pain?*
    - *What are you struggling with the most at home?*
    - *What do you wish you could most get help with?*

- Do you currently use any other additional resources to help manage your chronic pain?
    - *i.e. chiropractors, self-help groups, etc.*

- What have you wished was available to you at home to help you cope with your pain?
    - *medication assistance*
    - *reminder of various tools to choose from*
    - *assistance to set goals*
    - *guided meditations*

Now, I have some questions about the kind of technology you enjoy using, and not using.

- Do you use the Internet? How often do you use the Internet?
    - *How often do you use the internet for health-related reasons or to get answers to health-related questions?*

- What kinds of technology do you regularly use at home?
    - ☐ Laptops
    - ☐ iPad
    - ☐ iPhone/Android
    - ☐ Alexa/Google Home
    - ☐ Other

- Do you have a smart phone?
    - [yes] Do you use any tools or apps on your phone? What do you use apps them for?
    - [no] What went into your decision to not have a smart phone?

- Do you use any apps for health purposes? (e.g., those for chronic pain, tracking weight, exercise, diet, or other health behaviors)
    - *[yes] Can you tell me about some things you've done using health-related apps before?*
    - *[no] Would you consider using mobile apps for your health issues?*

- Think about the last piece of technology that you tried to use but did not like. What was it?
    - *Why did you like using that?*
    - *What didn't you like about using this?*

- How do you feel about at-home device that speaks to you, such as an Alexa of Google Home?
    - *[if they own one]: What do you like about it? What don't you like about it?*

- Have you ever been interested in an at-home, personal chatbot?
    - *What are your concerns about at-home robotics?*
    - *What do you think the benefits might be?*

<u>VACT-CP website demonstration</u>

Now, we're going to show you some of the activities we are designing for the VACT-CP website to do with Veterans at home. Feel free to discuss out loud your impressions while you interact with the module and virtual coach for the website, Coach Anne. This might include what you are doing in the moment, what you like, do not like, or would change. We want to get your opinion on different parts of each, so I will ask you questions after and sometimes during each demo. First, I will introduce you to VACT-CP website…

- **Provide participant with account information to log-in.**
- **Start VACT-CP interaction**

- **Query if not following think-aloud procedure**
    - *What are your general thoughts about Coach Anne responding to you like this?*
    - *How did you feel about the interaction style of responding to Coach Anne?*
    - *How do you feel about the questions Coach Anne is asking?*
    - *How would you feel about Coach Anne interacting with you at home?*
    - *What did you like about interacting with Coach Anne?*
    - *What did you not like about interacting with Coach Anne?*
    - *What would you change about this interaction? What would you change?*

Following VACT-CP website Demo: Qualitative Interview

Thank you for your feedback so far; the last thing we want to do is get some of your general and specific thoughts on VACT-CP website as a whole, and potential for future use. First….

VACT-CP website as a social interaction?

- What is your overall opinion of VACT-CP website?
    - *Was it what you expected?*
    - *How easy do you think it would be to use at home?*

- *Where you able to understand Coach Anne's voice?*
- *How would you feel about accessing the website weekly?*
- *What would you change about this interaction?*
- *How did you feel about the graphics and visual aspects?*

- How do you feel about Coach Anne's personality?

- How would you feel about Coach Anne continuing to have these kinds of conversations with you at home?

Perceived VACT-CP website Usability:

- What feature(s) do you like most and why?
  - *What part of the visuals did you most enjoy?*
  - *How did you feel about her audio skills?*
  - *What intervention exercises did you think would be helpful to you?*

- Which part(s) would you find less useful?

VACT-CP website as an At-Home Support for Chronic Pain

- What benefits do you see to having VACT-CP website with you at home?

- In what ways could we improve how you interact with VACT-CP website?
  - *What (else) would you suggest to make it easier to use (more user-friendly) or more inviting to use?*

- What might concern you about having VACT-CP website help you with at-home?
  - *[possible areas: privacy, data storage, general technology use concerns, etc.]*

- Would you be interested in having a VACT-CP website at home to help you with pain self-management in a future study? Why or why not?
  - *[if applicable] How do you think VACT-CP website might compare to your [most used tech for chronic pain]?*
