# Supplementary material for: An Online Acceptance and Mindfulness Intervention for Chronic Pain in Veterans: Development and Protocol for a Pilot Feasibility Randomized Controlled Trial
Source: JMIR Res Protoc. 2023 Mar 7;12:e45887. doi: 10.2196/45887 (PMC10031449; doi:10.2196/45887)
Supplement: Multimedia Appendix 4 [file resprot_v12i1e45887_app4.pdf]

PHASE 3: VACT-CP POST-INTERVENTION FEEDBACK INTERVIEW

GENERAL PROCEDURE

- Remind the Veteran that the entire feedback interview should take approximately half an hour. Obtain verbal consent to record audio file.
- Emphasize that their honest input is vital in helping to identify and prioritize needs, create solutions, and planning for services and future research use of the VACT-CP online program.
- Remember to thank the Veteran for their time, and ask if they have any questions at the end of the interview.

Thank you for finding the time to give me some feedback on the VACT-CP website today. Are you okay with me recording this feedback session, so we can make sure we don't miss anything?

Overview

- *First, let's start with the general research study itself. What led you to want to take part in this study to assess an online pain-management website?*
- *How do you feel about technology and its place in your healthcare?*
- *Were you interested in getting a therapeutic intervention for pain online?*

Coach Anne as an intervention guide:

- What is your overall opinion of the Veteran ACT for Chronic Pain online program?
  - *Was it what you expected?*
  - *How easy do you think it would be to use at home?*
  - *Did you complete all the modules? Why or why not?*
  - *How does it compare to other therapeutic interventions you have done for chronic pain?*
- *How do you feel about Coach Anne (personality, looks, etc?)*

Perceived VACT-CP Online Program Usability:

- *What feature(s) do you like most and why?*
    - *What part of the visuals/graphics did you most enjoy?*
    - *What intervention exercises did you think would be helpful to you?*

- *How did you feel about the 7 Modules? The additional Mindfulness module?*

- *How did you feel about the content (i.e., mindfulness, acceptance, values clarification)?*

- *How did you feel about different content options (e.g., metaphors, videos, values assessment)?*

- *In what ways did you find the modules and program might have benefited you? (mental health, chronic pain, quality of life?)*
    - *What if anything did you find useful to you?*
    - *Which part(s) did you find less useful?*

- *What benefits do you see to having the online program available to your or other Veterans?*

Suggestions for Improvement

- *In what ways could we improve how you interact with Coach Anne?*

- *What (else) would you suggest to make it easier to use (more user-friendly) or more inviting to use?*

- *What might concern you about using this online program?*
    - *[possible areas: privacy, data storage, general technology use concerns, etc.]*

- *Would you recommend this program to other Veterans struggling with chronic pain? Why or why not?*
