# Supplementary material for: An Online Acceptance and Mindfulness Intervention for Chronic Pain in Veterans: Development and Protocol for a Pilot Feasibility Randomized Controlled Trial
Source: JMIR Res Protoc. 2023 Mar 7;12:e45887. doi: 10.2196/45887 (PMC10031449; doi:10.2196/45887)
Supplement: Multimedia Appendix 5 [file resprot_v12i1e45887_app5.pdf]

SUMMARY STATEMENT

PROGRAM CONTACT: (Privileged Communication) *Release Date:* 03/27/2018

Revised Date:

Application Number: 1 IK2 RX002814-01A1

Principal Investigator

REILLY, ERIN

Applicant Organization: EDITH NOURSE ROGERS MEMORIAL VETERANS HOSPITAL

Review Group: RRD9
Career Development Program - Panel II

Meeting Date: 03/01/2018 *RFA/PA:* RX18-009
Council: MAY 2018
Requested Start: 06/01/2018

Project Title: Developing an Online Therapeutic Intervention for Chronic Pain in Veterans

SRG Action: Impact Score:135

Human Subjects: 30-Human subjects involved - Certified, no SRG concerns
Animal Subjects: 10-No live vertebrate animals involved for competing appl.
Gender: 1A-Both genders, scientifically acceptable
Minority: 1A-Minorities and non-minorities, scientifically acceptable
Children: 1A-Both Children and Adults, scientifically acceptable
Clinical Research - not NIH-defined Phase III Trial

Project Year	Direct Costs Requested
1	148,372
2	150,331
3	147,340
4	143,132
5	145,985
TOTAL	735,160

ADMINISTRATIVE BUDGET NOTE: The budget shown is the requested budget and has not been adjusted to reflect any recommendations made by reviewers. If an award is planned, the costs will be calculated by VA Office of Research and Development (ORD) staff based on the recommendations outlined in the BUDGET COMMENT section and any relevant ORD service-specific limitations.

SUMMARY OF DISCUSSION:
The Board met in Plenary Session and reviewed the above proposal considering all internal and external reviews. This document summarizes the major points of the discussion concerning the proposed project. In any further development of this project, the investigator should consider carefully all the issues reflected in this Summary of Discussion as well as the more detailed comments in the individual critiques.

GENERAL COMMENTS:
This is a very strong application. The proposed project is significant, as development of interventions to facilitate self-management for Veterans with chronic pain is of great importance. The aims of the project address an important issue for the Veteran Affairs (VA) and for Veterans. The mentoring team is well-established and successful. Resources for training and carrying out the proposed research project are identified, and the letters of support are strong. The applicant has the requisite background of training, experience, and importantly, dedication to a productive career that predict success.

Additionally, the applicant was highly responsive to reviewer and committee concerns. The applicant added letters from the appropriate personnel to show high feasibility, and effectively addressed recruitment concerns. In addition, she added two additional consultants that strengthen the proposal and she better explained many of the study logistics (including her engagement in mentor-related activities). It is clear that she is receiving strong mentorship.

SUGGESTIONS:
- The applicant may consider broadening the thematic approach for Study Aim 1. It may be beneficial to collect a broad range of expert opinion, rather than limit it to specific themes.
- The second training goal may be too specific and may be broadened to gain more experience in developing the proposed interventions.
- It remains unclear whether participants may be engaged in other treatments during the study.
- ACT can be a challenging intervention to implement. The applicant indicates that the proposed intervention will be interactive, but it remains unclear exactly what this would look like. Additional clarification would be helpful.
- The applicant may consider engaging providers and Veterans in a discussion about safety related issues (the help menu button) during Phase 1.

COMMENTS ON THE BUDGET:
None.

DESCRIPTION (provided by applicant):
Chronic pain is a serious concern that disproportionately affects Veterans compared to the general public; Veterans are diagnosed with CP at particularly high rates (47 – 56%) with a 40% greater rate of severe pain than non-Veterans. Veterans with chronic pain face numerous negative functional outcomes, including decreased ability to complete daily work activities, less social support from and closeness with family members, increased chronic health conditions (e.g., cancer, heart disease), and higher mortality compared to Veterans without chronic pain. Given these concerns, there is an urgent need for innovative and integrative approaches for non-medical pain self-management management. Despite the critical importance of effective pain self-management programs, many Veterans with CP do not engage in pain self-management programs. In order to improve Veterans' quality of life, it is important to develop and evaluate innovative, accessible, evidence-based interventions for managing CP. One approach with over twenty years of efficacious treatment for chronic pain is Acceptance and Commitment Therapy for Chronic Pain (ACT-CP). ACT is a well-established VA-approved approach to chronic pain management, and focuses on committing to behavior change that reflects personal values, leading to significant improvement in life functioning. ACT- CP is associated with substantial improvements in social/work functioning and decreased pain-related medical visits, as long as three years following treatment. For adults with chronic pain, technology-assisted ACT treatment leads to

reduced self-reported pain levels and improved health via changes in value-aligned behaviors and mindfulness. The use of interactive technology-assisted ACT treatment is acceptable and efficacious; however, no ACT for chronic pain online treatment exists for Veterans. We thus propose a three-phase development, intervention usability and feasibility, and RCT pilot to create a virtual ACT intervention for CP (VACT-CP) for Veterans. VACT-CP will utilize a personalized, social interface to address pain-related distress and functional difficulties of chronic pain (e.g., avoidance, reactivity), using an online Embodied Conversational Agent (ECA) that will walk Veterans through eight weeks of treatment. Studies suggest that the use of such ECAs can increase online-treatment motivation and feedback, resulting in increased treatment compliance and utilization, physical functioning (e.g., increased physical activity and diet fidelity), and client-goal achievement. The primary outcomes for this project will be to (1) develop the VACT-CP user system using feedback from mental health and other clinical professionals treating chronic pain (n = 10), (2) pilot the usability and feasibility of the through iterative usability development and Veteran feedback (n = 12 - 15), and (3) explore the impact of the VACT-CP system in terms of user- experience, functional outcome improvement, and quality of life measures (n = 40).

PUBLIC HEALTH RELEVANCE:
Non-pharmacological chronic pain treatment is an urgent need for Veterans; this proposed project will increase access to evidence-based, non-pharmacological chronic pain treatment by providing Veterans with an acceptance and commitment based online treatment intervention aimed at reducing pain-related distress and improving daily functioning. The VHA has committed to exploring innovative treatments for Veterans with chronic pain, and this project would align with VISN 1 Integrated Pain System strategic goals and Standards of Pain Management of 1) pain assessment and treatment and 2) evaluation of outcomes and quality of pain management. This work will fill a substantial gap in VHA pain services by developing an at-home technology-assisted treatment for chronic pain that can lead to substantially increased access to effective chronic pain treatment for Veterans.

CRITIQUE 1

CRITIQUE (Applicant, Mentor, and Training Program):

Applicant: This is the second time this reviewer has seen this application. The applicant is a strong candidate, and has clear potential to develop as an independent scientist-practitioner as evidenced by her training and research experience.

The applicant has provided several strong letters of support, which reflect her qualifications as a clinician, her technological skills, and familiarity with developing manualized treatments, history of productivity (publications and grant funding), commitment to research and excellence, and strong moral character. In addition, the applicant has addressed reviewer concerns. Based on her thoughtful and skillful responses, it is clear that she is receiving strong mentorship from her team.

Mentor/s: The applicant has assembled a strong team of mentors with whom she has familiarity and a history of productive research endeavors, and added two new consultants, one with whom has expertise in testing strategies for the implementation of internet-based self-care interventions (Hermes) and the other who is the National Program Manager in the Office of Mental Health Services & Suicide Prevention, directing VA's program of web-based self-help (Greene). Mentors have well-documented histories of successful training and mentorship. All content and expertise appear very strong.

The applicant has responded sufficiently to reviewer concerns of potential over commitment to mentor research projects during the CDA funding period, stating that her effort would be minimal (about 5-15% weekly) and focused on addressing her training goals and manuscript writing.

Training Program: The proposed training plan is comprehensive; didactic courses are planned, mentor-applicant interactions are generally well-described, and research monitoring is sufficiently detailed. The training program is well-designed to lead to the applicant's growth and development as a VA researcher. The applicant added a new aim in response to reviewer recommendations to increase training and VA culture and leadership.

Summary of Training Proposal: The applicant's training goals include:
- Developing an expertise in clinical trial methodology.
- Increasing skills in iterative usability and feasibility testing for technology-assisted interventions.
- Strengthening experience, knowledge, and skill in ACT treatment for chronic pain.
- Increasing her knowledge in advanced statistics relevant to analyzing treatment research.
- Increase training in VA culture and leadership related to mental health and chronic pain research.

The applicant has adequately addressed prior reviewer concerns including clarification of the time frame and time engaged in mentor research: the applicant has reduced her time commitment from 40% to 15% in Years 1-2. The applicant has further outlined the specific roles as they align with the training goals, and confirmed that the applicant will not function as a research assistant for these projects. Additionally, the training plan now includes grant-writing opportunities, leadership courses and a new training goal to become more embedded in VA.

CRITIQUE (Scientific Merit—Strengths, Weaknesses, & Recommendations):

Significance:
Strengths:
- Effective, chronic pain interventions are of immediate need for the Veteran population. The stepped-care model (currently adapted by the VA) emphasizes self-management of chronic pain in Step 1. Programs that can assist Veterans with chronic pain conditions and facilitate engagement in their own pain management are needed. This project seeks to address a gap in existing chronic pain treatments by modifying an empirically supported treatment and developing and implementing an innovative online treatment delivered by an embodied conversational agent.
- Access to care is a challenge for Veterans. This project seeks to develop a technology that will not require direct interaction with a human being that requires an FTE. This could improve access to care for Veterans who are interested in engaging in the intervention. Thus, potential findings may have an important role to the health and healthcare of Veterans.
- The proposal is well-written and well-organized. The applicant has clearly worked closely with her mentors in assembling this CDA-2 proposal.

The applicant was very responsive to reviewer recommendations.

Specific Applicant Response to Concerns:
- The applicant clarified that a wait list control will be designated as the control group. The applicant states that the wait list control will not; however, constitute "no treatment" as Veterans will receive a handout with information about different available support options for Veterans with chronic pain and the local facility. Of note, it is appreciated that the applicant included a discussion about non-inferiority trial data in the research plan, as this addresses some concerns about the waitlist control option and trial design. Additionally, the applicant further explained the relevance of the ACT web-based tobacco cessation intervention, which demonstrates receptivity of web based delivery of ACT, albeit in a different population (PTSD/smoking).
- The applicant briefly addressed concerns about inclusion criteria by indicating that MSK and joint pain (which are not differentiated) are the most likely to benefit from psychological and behavioral interventions. The applicant clarified the no co-occurring pain conditions will exclude study participation.

- The applicant addressed concerns about the inclusion of Veterans without access to high speed internet. To address these challenges, the applicant added an alternative participation option to allow Veterans to access and go through the intervention each week within a secure space at the Bedford VA with a VA-provided tablet. She indicated that Veterans would also be provided with free Wi-Fi locations outside of their home such as libraries, CBOCs, etc. The applicant's mentors will also provide ideas and recommendations for this barrier for the current project and for future merit applications.
- The applicant reports in the response letter that she will implement a two-fold process during Phase 3 to address suicidal/homicidal ideation assessment. A research assistant will call participants at weeks 3 and 6 to assess for safety concerns. She also mentions that the intervention itself will include a mechanism in the program (from the main menu option) that connects Veterans to the VA crisis line and provides contact information for the PI. This new information is addressed in the protocol, (Adaptation to Online Platform). However, it remains unclear how the avatar will (if at all) respond to those who may present with SI/HI who may not necessarily be seeking assistance. Will individuals be required to access the help menu or will the avatar be able to detect and respond to a Veteran expressing concerning behavior/phrases, etc.?

It is noted that the applicant has consulted with several of her mentors about potential safety concerns online, and provided their expertise; this reviewer feels that safety concerns will be addresses in an effective and appropriate manner.

The applicant and mentors may additionally consider:
- Asking providers about safety related issues (SI/HI) during Phase 1. Since this group will be comprised of mental health providers, feedback about usability and response of the program to SI/HI concerns could be of great benefit. This may also be helpful to assess with Veterans in Phase 2. That is not just usability (i.e., locating the button), but asking whether or not a Veteran might use the option if they are feeling suicidal. It also warrants other questions, such as will these sessions be monitored by live providers during the trial? If so, the applicant and her mentors may wish to consider steps they would take to maximize safety of participants during times when interacting with the avatar. Participants may not be willing to call a hotline, so other immediate interventional strategies may be required. Again, the applicant's mentors/consultants have substantial expertise in suicide prevention, and can provide guidance to the applicant as needed.
- May wish to better define chronic pain. Inclusion criteria states a score of four or higher at three separate VA outpatient visits in the past year. A definition of chronic pain that includes duration may be helpful (i.e., experienced for 3 months or 6 months).

The applicant provided specific hypotheses, which are well articulated and thought out.

Approach: The proposed research study has three aims:
- To modify ACT for chronic pain to a socially-interactive online platform with solicitation of feedback from treatment providers (n=10).
- Assess the feasibility and usability of Veteran Acceptance and Commitment Therapy for Chronic Pain (VACT-CP) and an avatar with Veterans (n=12-15) via field-based iterative usability testing.
- Conduct a pilot feasibility RCT (n=20) using VACT-CP compared to a wait-list control (n=20).

Overall, the proposed methods are appropriate for stage 1 behavioral treatment development. The research is highly innovative using state-of-the-art treatment delivery technology.

Environment: The proposed academic environment is idea for the completion of the proposed project. As stated by the applicant, the proposed work will capitalize on the resources of the Social and Community Reintegration Research (SoCRR) program, a Rehabilitation Research & Development-funded Research Enhancement Award Program (REAP) at the Edith Nourse Rogers Memorial Veterans Hospital. The applicant has resources available from UMASS Medical School and Boston

University, and is embedded as a postdoctoral trainee in both The Center for Healthcare Organization and Implementation Research (CHOIR) and The New England Mental Illness Research Education and Clinical Center (MIRECC), and has access to their resources in support from The Psychology Training Program and CHOIR postdoctoral fellowship program. In addition, the applicant has assembled a collaborative team of investigators, already working collaboratively on projects. Multiple letters of support detail a high level of enthusiasm for the candidate's background, experience, skill and commitment to the project and VA scientist. The facility director supports the project and will provide space. The applicant has adequate resources to carry out this project

Feasibility: The applicant has successfully addressed prior concerns about feasibility. Specifically, she clarified that the ACT for tobacco cessation program (similar to the current proposal) conducted by the applicant and her mentor have met all IRB and privacy standards. In addition, she added two consultants, one of whom has conducted research for online treatment with Veterans and another who is providing national level support for the project as the National Program Manager in the Office of Mental Health Services & Suicide Prevention. She has also consulted with her local IRB chair and received assurance that the proposed research would follow local IT facility procedures for both IRB and security issues (a letter from Dr. John Wells, IRB chair was provided).

The applicant also consulted with their local data administrator to verify that 2605 Veterans are enrolled in the Bedford VA with chronic non-cancer pain, suggesting that the recruitment goal of 40 Veterans is feasible. They also extended recruitment for Phase 3 to 15 months to increase feasibility. Finally, the applicant re-iterated, and clarified that the study team will iteratively address usability issues until the system is ready for testing in the feasibility pilot RCT in Phase 2. Thus, there should be no anticipated concerns about feasibility for a Merit Review proposal.

Overall Evaluation (Applicant/Mentor(s)/Training and Scientific Merit):
This is a very strong application. Development of interventions to facilitate self-management for Veterans with chronic pain is of great importance. The aims of the project address an important issue for the VA and for Veterans. The mentoring team is well-established and successful. Resources for training and carrying out the proposed research project are identified, and the letters of support are strong. The applicant has the requisite background of training, experience, and importantly, dedication to a productive career that predict success.

Ethical/Safety Issues: None.

OTHER CONSIDERATIONS:

Clinical Relevance: Highly clinically relevant.

Budget: No concerns.

Other Issues: No Comment.

CRITIQUE 2

CRITIQUE (Applicant, Mentor, and Training Program):

Applicant: Dr. Reilly is a productive researcher with a background well suited to her proposed work. Her background in ACT and in virtual and other technology based interventions would serve her well in meeting her research goals. She is relatively new to VA, but in the revised application has added additional training activities to learn more about VA and Veterans. She has very strong letters of recommendation, describing her as exceptionally productive across several settings.

Mentor/s: Excellent team of mentors and consultants whose expertise across research, ACT, technology, and conducting RCT's maps on well to Dr. Reilly's training goals. Several of the letters highlighted how development and application of the intervention will be feasible. Adding Dr. Carolyn Greene, who has worked on many VA virtual self-help programs, as a consultant strengthens the proposal.

Training Program: Dr. Reilly's training goals are:

- Assisting in development understanding of clinical trials methodology.
- Increasing knowledge of iterative usability and feasibility testing for technology-assisted interventions.
- Strengthening expertise in chronic pain clinical treatment.
- Increasing knowledge in advanced statistics relevant to the analyzing treatment research.
- Training in VA culture, leadership, and Veteran-centered chronic pain research.

The training program is well suited to the applicant's training goals. The addition of the goal to train in VA culture and leadership strengthens the proposal.

The second goal regarding increasing skills in iterative usability and feasibility testing seems like it could be expanded to more broadly cover development and evaluation of virtual interventions since this is these are the primary grant activities and among Dr. Reilly's long term career goals.

CRITIQUE (Scientific Merit—Strengths, Weaknesses, & Recommendations):

Significance: Innovative behavioral interventions for treating chronic pain are very important to VA. A virtual program that Veterans can complete from home has potential to help a large number of Veterans.

Weaknesses: None noted.

Approach: The applicant was highly responsive to the last round of critiques. In particular, the revised application is stronger in describing logistics such as how the intervention will be developed and how approvals will be obtained.

Generally, the intervention development and assessment process proposed is appropriate for the study goals. Some concerns detailed below:

Study 1: Would a thematic approach adequately capture the diversity of opinions that may be presented? Is the goal to get a uniform set of providers or ones with a diversity of expertise? If a diversity of perspectives is the goal, would unique but important opinions be lost by using an approach that looks for common themes?

Study 3: Many of the potential participants will arrive at the pain clinic with the goal of accessing in person pain care. If they agree to be in the study, they will get a self-help program or a waitlist control. This raises a few questions.

If participants would like to access a clinician (for reasons other than being in crisis) during the study, how would they go about doing so?

Are there any exclusions related to treatment for other comorbidities? For example, can participants be completing psychotherapy for PTSD at the same time as they are in the study?

It would be helpful if the proposal explicitly addressed use of opioids. It appears opioids are ok as long as there is no opiate use disorder. Are there any other concerns regarding participants on opioids?

Environment: Very strong research environment.

Feasibility: The revised application gives a far stronger justification for the study's feasibility. Letters from the local facility, collaborators, and national VA leaders in virtual intervention strengthen confidence in the study's feasibility.

Overall Evaluation (Applicant/Mentor(s)/Training and Scientific Merit):
Dr. Reilly is a well-qualified applicant with clearly identified goals, a strong mentor team, and well suited training plan. Her background in technology-based interventions and in ACT makes her an ideal candidate for the study she proposes. The proposed intervention is exciting as self-help interventions for pain have the potential to help many Veterans. A few concerns about the study design remain, particularly regarding the use of a waitlist control group for a treatment seeking sample of pain patients.

Ethical/Safety Issues: No comment.

OTHER CONSIDERATIONS:

Clinical Relevance: The intervention has the potential to reach many Veterans with a common and highly impairing condition.

Budget: No concerns.

Other Issues: None noted.

CRITIQUE 3

CRITIQUE (Applicant, Mentor, and Training Program):

Applicant: The applicant is currently a post-doctoral fellow. She graduated with her PhD in 2016. Although quite junior as an investigator, her productivity is high with many publications and leadership as PI or co-PI on small grant projects. She has experience administering ACT interventions with other populations and has been conducting some other research within the VA. Her letters of support were enthusiastic and support her strong performance.

Mentor/s: The mentors are well-suited for this project. Dr. Kelly, Associate Professor in Psychiatry and director of the VISN New England MIRECC and treats Veterans with chronic pain at the Bedford VA, She specializes in ACT interventions with several funded projects in the area. She also has an established relationship with the applicant. The other mentors have specific roles necessary for fulfilling training or research goals (such as training in usability testing and statistical techniques). The majority of mentors have been working together with the applicant. The applicant addressed some previous critiques adequately from the last cycle, reducing the weekly meeting schedule with four mentors and specifying how meetings with out of town mentors would occur.

Training Program: From the last cycle, the training plan was amended to address the reviewer critique by adding a VA specific career development training goal and will include membership on national VA committees on chronic pain, leadership courses, and integration in STEM research and academic groups. The training plan is comprehensive and thoughtfully constructed to meet her training needs.

CRITIQUE (Scientific Merit—Strengths, Weaknesses, & Recommendations):

Significance: Chronic pain is prevalent and disabling among Veterans. Acceptance and commitment therapy is a recommended behavioral treatment for depression at the VA and offering it to via web would provide access for many Veterans.

Approach: The purpose of this study is to test the feasibility of a technology-assisted acceptance and commitment therapy (ACT) intervention for Veterans with chronic pain. ACT is an effective approach for Veterans but many practitioners are not trained on how to give this treatment and people suffer from lack of access due to travel constraints. There are three aims to the proposal-development of the online platform, feasibility and usability testing of the intervention, and then a pilot feasibility test of the intervention. The methods are well-laid out and are in an appropriate scope for this project. The applicant adequately addressed concerns from the last critique, clarifying the inclusion and exclusion criteria, adding in more specific data analyses, and adding an alternative option for Veterans to participate who do not have internet at home, by providing a laptop at the Bedford VA.

Environment: Given her mentors' expertise in clinical trial development and ACT (particularly Dr. Kelly and Dr. Heapy), the environment is ideal for the applicant to meet her goals.

Feasibility: The project appears feasible now that the recruitment timeline has been extended.

Overall Evaluation (Applicant/Mentor(s)/Training and Scientific Merit):
This is a strong proposal from Dr. Reilly and team, particularly because it is a project that is both significant and innovative and provides an ideal training environment for the applicant. The project is clear and is of appropriate scope for this award. In addition, it would provide fundamental pilot data on which to further test the technology-assisted ACT intervention in a larger trial which is a main goal. The aims are logical and build upon each other appropriately. The applicant has built a strong research and training project, has a stellar team, and is poised to meet her goals with these plans.

Ethical/Safety Issues: The human subjects section was comprehensive and appropriate.

OTHER CONSIDERATIONS:

Clinical Relevance: The potential to impact chronic pain and functioning in Veterans is high because development is theoretically-grounded, borne out of the clinical use of ACT, and actively utilizes Veteran and provider feedback to design the intervention.

Budget: No issues.

Other Issues: No comment.

MEETING ROSTER

Career Development Program - Panel II
Rehabilitation Research and Development Parent IRG
Office of Research & Development
RRD9
03/01/2018

CHAIRPERSON(S)

WECHT, JILL M., EDD
RESEARCH ASSOCIATE/PRINCIPAL INVESTIGATOR
SPINAL CORD DAMAGE RESEARCH CENTER
JAMES J. PETERS VA MEDICAL CENTER (BRONX)
PROFESSOR OF MEDICINE AND REHABILITATION MEDICINE
MOUNT SINAI SCHOOL OF MEDICINE
BRONX, NY 10468

MEMBERS

ACIERNO, RONALD E., PHD *
DIRECTOR OF PTSD CLINICAL TEAM
CHARLESTON VA MEDICAL CENTER
PROFESSOR
DEPT OF PSYCHIATRY AND BEHAVIORAL SCIENCES
MEDICAL UNIVERSITY OF SOUTH CAROLINA
CHARLESTON, SC 29403

BAHRAINI, NAZANIN HADAEGHI, PHD *
EDUCATION DIRECTOR
VA EASTERN COLORADO HEALTH CARE SYSTEM
VISN 19 MIRECC
DENVER, CO 80220

BAYLEY, PETER J., PHD *
RESEARCH HEALTH SCIENCE SPECIALIST
VA PALO ALTO HEALTH CARE SYSTEM
PALO ALTO, CA 94304

BEGUM, MOMOTAZ PHD *
ASSISTANT PROFESSOR
DEPARTMENT OF COMPUTER SCIENCE
UNIVERSITY OF NEW HAMPSHIRE
DURHAM, NH 03824

FISCHER, ELLEN P, PHD *
RESEARCH HEALTH SCIENTIST
LITTLE ROCK VA MEDICAL CENTER
ASSOCIATE PROFESSOR
DEPARTMENT OF PSYCHIATRY
UNIVERSITY ARKANSAS FOR MED SCIENCES
LITTLE ROCK, AR 72204

FISHER, JONATHAN A.N., PHD *
ASSISTANT PROFESSOR
DEPARTMENT OF PHYSIOLOGY
NEW YORK MEDICAL COLLEGE
VALHALLA, NY 10595

GOODMAN, MARIANNE, MD *
ASSOCIATE DIRECTOR OF EDUCATION
MENTAL ILLNESS RESEARCH
EDUCATION AND CLINICAL CENTERS
JAMES J PETERS VA MEDICAL CENTER
BRONX, NY 10468

HACKNEY, MADELEINE E., PHD *
HEALTH SCIENTIST
ATLANTA VA MEDICAL CENTER
ASSISTANT PROFESSOR OF MEDICINE
EMORY UNIVERSITY
DECATUR, GA 30033

KEAN, JACOB T., PHD *
RESEARCH SPEECH PATHOLOGIST
HSR&D CIEBP
RICHARD L. ROUDEBUSH VA MEDICAL CENTER
ASSIST PROF, DEPT OF PHYSICAL MED & REHAB
INDIANA UNIVERSITY SCHOOL OF MEDICINE
INDIANAPOLIS, IN 46202

KING, LAURIE PT, PHD *
RESEARCH INVESTIGATOR
VA PORTLAND HEALTH CARE SYSTEM
ASSISTANT PROFESSOR OF NEUROLOGY
OREGON HEALTH & SCIENCES UNIVERSITY
PORTLAND, OR 97239

LEPAGE, JAMES, PHD *
ASSOCIATE CHIEF OF STAFF FOR RESEARCH AND DEVELOPMENT
DALLAS VA MEDICAL CENTER
DALLAS, TX 75216

MURPHY, SUSAN L., SCD *
RESEARCH HEALTH SCIENCE SPECIALIST
ANN ARBOR VA HEALTH CARE SYSTEM GRECC
ASSISTANT PROFESSOR
PHYSICAL MEDICINE AND REHABILITATION DEPARTMENT
UNIVERSITY OF MICHIGAN
ANN ARBOR, MI 48105

NAYLOR, JENNIFER C, PHD *
PSYCHOLOGIST
DURHAM VA MEDICAL CENTER
ASSISTANT PROFESSOR
DEPT OF PSYCHIATRY
DUKE UNIVERSITY MEDICAL CENTER
DURHAM, NC 27705

NOCERA, JOE ROBERT, PHD *
HEALTH SCIENCE SPECIALIST
ATLANTA VA MEDICAL CENTER
ASSISTANT PROFESSOR
DEPARTMENT OF NEUROLOGY
EMORY UNIVERSITY SCHOOL OF MEDICINE
ATLANTA, GA 30322

NORMAN, SONYA, PHD *
PSYCHOLOGIST
SAN DIEGO VA MEDICAL CENTER
ASSOCIATE CLINICAL PROFESSOR
UNIVERSITY OF CALIFORNIA SAN DIEGO
SAN DIEGO, CA 92108

PHILIP, NOAH STEPHEN, MD *
DIRECTOR, PSYCHIATRIC NEUROMODULATION
PROVIDENCE VA MEDICAL CENTER
ASSOCIATE PROFESSOR
PSYCHIATRY AND HUMAN BEHAVIOR
ALPERT MEDICAL SCHOOL OF BROWN UNIVERSITY
PROVIDENCE, RI 02912

SHINN, JENNIFER A, PHD *
ASSOCIATE PROFESSOR AND CHIEF OF AUDIOLOGY
UNIVERSITY OF KENTUCKY
LEXINGTON, KY 40536

SLOAN, DENISE M., PHD *
STAFF PSYCHOLOGIST, PTSD, BSD
BOSTON VA HEALTHCARE SYSTEM
PROFESSOR
DEPARTMENT OF PSYCHIATRY
BOSTON UNIVERSITY SCHOOL OF MEDICINE
BOSTON, MA 02130

TESKE, JENNIFER ANN, PHD *
ASSISTANT PROFESSOR, NUTRITIONAL SCIENCES
THE UNIVERSITY OF ARIZONA
TUCSON, AZ 85721

TURNER, AARON P., PHD *
PSYCHOLOGIST
DIRECTOR, REHABILITAITON PSYCHOLOGY
PUGET SOUND VA HEALTHCARE SYSTEM
HARBORVIEW MEDICAL CENTER
SEATTLE, WA 98108

WRAY, D. WALTER WALTER, PHD *
RESEARCH INVESTIGATOR
SALT LAKE CITY VAMC
ASSOCIATE PROFESSOR
DEPARTMENT OF MEDICINE
UNIVERSITY OF UTAH
SALT LAKE CITY, UT 84148

SCIENTIFIC REVIEW OFFICER

GROER, SHIRLEY, PHD
SCIENTIFIC PROGRAM MANAGER
DEPARTMENT OF VETERANS AFFAIRS
OFFICE OF RESEARCH AND DEVELOPMENT
REHABILITATION RESEARCH AND DEVELOPMENT SERVICE
WASHINGTON, DC 20420

* Temporary Member. For grant applications, temporary members may participate in the entire meeting or may review only selected applications as needed.

Consultants are required to absent themselves from the room during the review of any application if their presence would constitute or appear to constitute a conflict of interest.
